# Adjacent Nucleotide Dependence in ncRNA and Order-1 SCFG for ncRNA Identification

**DOI:** 10.1371/journal.pone.0012848

**Published:** 2010-09-28

**Authors:** Thomas K. F. Wong, Tak-Wah Lam, Wing-Kin Sung, Siu-Ming Yiu

**Affiliations:** 1 Department of Computer Science, The University of Hong Kong, Hong Kong, Special Administrative Region, People's Republic of China; 2 School of Computing, National University of Singapore, Singapore, Singapore; Aarhus University, Denmark

## Abstract

**Background:**

Non-coding RNAs (ncRNAs) are known to be involved in many critical biological processes, and identification of ncRNAs is an important task in biological research. A popular software, Infernal, is the most successful prediction tool and exhibits high sensitivity. The application of Infernal has been mainly focused on small suspected regions. We tried to apply Infernal on a chromosome level; the results have high sensitivity, yet contain many false positives. Further enhancing Infernal for chromosome level or genome wide study is desirable.

**Methodology:**

Based on the conjecture that adjacent nucleotide dependence affects the stability of the secondary structure of an ncRNA, we first conduct a systematic study on human ncRNAs and find that adjacent nucleotide dependence in human ncRNA should be useful for identifying ncRNAs. We then incorporate this dependence in the SCFG model and develop a new order-1 SCFG model for identifying ncRNAs.

**Conclusions:**

With respect to our experiments on human chromosomes, the proposed new model can eliminate more than 50% false positives reported by Infernal while maintaining the same sensitivity. The executable and the source code of programs are freely available at http://i.cs.hku.hk/~kfwong/order1scfg.

## Introduction

A non-coding RNA (ncRNA) is a RNA molecule which is not translated into a protein. It has been shown to be involved in many biological processes [1]–[3]. The number of ncRNAs was underestimated before, but recently some databases reveal over 200K ncRNAs [4] and more than 1,300 ncRNA families [5]. Large discoveries of ncRNAs and their families show the possibilities that ncRNAs may be as diverse as protein molecules. Identifying ncRNAs is an important problem in biological study.

Since ncRNAs do not translate into protein, it is more difficult to detect and identify these molecules in laboratories. Also, the process is usually time-consuming and expensive. On the other hand, it is known that the structure (both the primary and the secondary structure) of an ncRNA molecule usually plays an important role in its biological functions. Computational approaches provide an alternative to identify potential ncRNA candidates. In general, computational methods work as follows (e.g. [6]–[8]). For each ncRNA family, we build a structural model. Then, scan the entire genome and align every region of the genome with the structural model of the family. The region which results in high score will be regarded as a potential member of the family. The structural model is the core of the computational method as it should capture the characteristics of a given ncRNA family and should be powerful enough to distinguish members in the family from other sequences.

To capture both the primary and secondary structure of an ncRNA molecule, a popular method is to use stochastic context free grammar (SCFG). This was first suggested by [9]. Examples of computational tools that are based on SCFG model for predicting ncRNA family members are tRNAScan-SE [10], Infernal [11], [12] and RSEARCH [13]. Among these tools, Infernal is the most successful tool and exhibits high sensitivity. Infernal was used to develop Rfam [5], one of the most comprehensive and popular ncRNA databases. The application of Infernal was often limited to small suspected regions. Using a small computer cluster, we conducted an experiment of using Infernal to identify ncRNAs on a human chromosome level. The sensitivity remains very high, yet Infernal gives quite many false positive candidates. In our experiment, we select all the human chromosomes. There are about 8,000 known ncRNA members in these chromosomes; Infernal reports more than 45,000 candidates. It is likely that many of these are false positives. To further justify this observation, we generate random DNA sequences and use Infernal to scan through the sequences. Although it is unlikely to have many real ncRNAs in these random sequences, Infernal still reports quite a number of candidates (see the Results and Discussions Section for more details). Due to large number of false positives, it is time-consuming and expensive to verify each of predicted candidates in order to identify the true positives. It also reveals that the SCFG model may not be powerful enough to differentiate the false positives from the real ncRNA members.

After studying this issue in details, we found that the SCFG model used in all these software tools does not consider the dependence between the nucleotides in the ncRNA sequence. However, the dependency of adjacent nucleotides does affect the stability of the pairing structure of an ncRNA as the free energy of a structure depends on what the adjacent nucleotides are. Most of the recent free-energy models capture this dependence when computing the free energy of a given secondary structure [14]–[16]. It is likely that considering adjacent nucleotide dependency when identifying ncRNAs can increase the accuracy.

In this study, the objective is two-fold. First, we want to investigate if the signal based on the dependence of adjacent nucleotides in a human ncRNA sequence is strong enough for us to differentiate real ncRNAs from false positives. Recently, [17] has independently raised a similar concern on the nucleotide dependence in ncRNA sequences and conducted a primitive study on a relatively smaller AT-rich Dictyostelium discoideum genome. Second, we try to incorporate adjacent nucleotide dependence into the SCFG model, which is technically non-trivial. We propose a new order-1 SCFG model and based on this model, we develop a more effective tool to find ncRNAs. The aim is to maintain high sensitivity while removing most false positives.

### The results

(1) We conduct a systematic study on the dependence of adjacent nucleotides of the known members in all ncRNA families and compare the dependence with that of the candidates reported by Infernal, but not found in known databases (although there should be real ncRNAs among these candidates, most of them will probably be false positives). We examine the dependency of adjacent single bases as well as adjacent base pairs. We found that the adjacent nucleotide dependence of those candidates reported by Infernal is much smaller than that of the known members of an ncRNA family (See Table 1 for more details). This motivates us to use the nucleotide dependence of the candidates to distinguish false positives from real human ncRNAs. (2) We enhance the SCFG model to take into account the dependency of adjacent nucleotides and come up with a new order-1 SCFG model. We provide an algorithm to build the production rules of the order-1 SCFG for a given set of ncRNA sequences in a family. We implement our model for all the known human ncRNA families and use it to filter the results from Infernal. From our experiments on the same set of chromosomes, our approach can reduce the total number of candidates by more than 50% reported by Infernal while maintaining the same sensitivity.

**Table 1:** Analysis of the adjacent nucleotide or base pair dependency of known ncRNAs and other candidates reported by Infernal.

			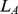			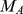		
Family	All known human members in Rfam	Other candidates reported by Infernal	All known members of all species ( 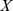 )	Other candidates ( 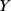 )	Difference ( 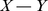 )	All known members of all species ( 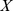 )	Other candidates ( 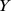 )	Difference ( 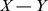 )
RF01382	0	8985	2.7	0.5	2.2	8.85	2.4	6.44
RF00017	0	4704	1.02	0.71	0.32	2.14	1.45	0.7
RF00037	33	1552	0.74	0.72	0.02	3.96	2.12	1.84
RF00825	0	1415	1.64	0.24	1.4	5.14	1.69	3.45
RF00711	3	1019	1.32	0.31	1.01	4.4	1.63	2.77
RF00736	18	1012	1.23	0.35	0.88	4.67	1.43	3.24
RF00651	2	913	1.37	0.52	0.85	5.44	2.21	3.23
RF00647	0	906	1.38	0.44	0.95	5.98	2.16	3.81
RF00464	2	887	1.13	0.31	0.82	5.15	1.92	3.23
RF00031	20	687	1.28	0.57	0.71	2.6	2.06	0.54
RF00876	32	613	0.81	0.5	0.31	5.73	2.15	3.58
RF00693	5	548	1.14	0.53	0.61	4.35	1.67	2.68
RF00951	744	521	0.44	0.72	−0.28	1.41	2.06	−0.65
RF00131	3	485	1.71	0.3	1.41	6.24	4.04	2.2
RF00001	431	287	0.66	0.78	−0.11	1.23	1.83	−0.6
RF00646	2	270	1.52	0.41	1.11	6.02	3.15	2.87
RF00027	12	247	1.16	0.43	0.74	5.57	3.16	2.42
RF00685	0	243	1.06	0.51	0.55	4.41	2.94	1.47
RF00239	3	192	1.83	0.57	1.26	4.99	2.36	2.63
RF00140	0	190	2.31	0.65	1.66	3.34	1.46	1.89
		**Average**	**1.32**	**0.5**	**0.82**	**4.58**	**2.19**	**2.39**

The second and the third columns show the number of known members and other candidates reported by Infernal for some families. The column 4,5,6 (or 7,8,9) show the comparison of the dependence of adjacent nucleotides along single-stranded regions (or adjacent base pairs along stacking pair regions) between all known ncRNA members (i.e. full members) in Rfam and the other candidates reported by Infernal of each family. Larger value of 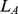 (or 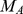) indicates the higher level of dependence between the adjacent single-stranded columns (or paired columns) within the multiple sequence alignment 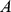. The table lists the top 20 families with the highest number of candidates reported by Infernal. As we can see, in most of the cases, the level of adjacent dependence along both single-stranded regions and stacking pair regions in known ncRNAs is higher than that in other candidates reported by Infernal. The % of difference between the dependence levels with respect to the values of other candidates are 164% and 109% along single-stranded regions and stacking pair regions respectively. This provides evidence to support our conjecture that the adjacent dependence in human ncRNA molecules should be useful to distinguish real ncRNAs from false positives.

## Results and Discussion

### Scanning chromosomes by Infernal

We selected all human chromosomes and used Infernal to scan through each entire chromosome to locate possible ncRNA members. We check the reported candidates against the listed ncRNAs of each corresponding human chromosome in Rfam 9.1 database. Rfam was first developed using Infernal and it was then curated and maintained by a group of researchers in Wellcome Trust Sanger Institute from time to time. This ncRNA database has now becoming one of the most comprehensive and trustable ncRNA database. We found that apart from the known members in these chromosomes, Infernal reported many other candidates. There are around 8,000 known ncRNAs in all human chromosomes. Infernal reports more than 45,000 candidates. The second and the third columns in Table 1 show the number of known members and other candidates reported by Infernal for some families. Some of these other candidates, which are not known ncRNAs, may be novel ncRNAs. However, there should not be that many novel ncRNAs, so most of them are probably false positives.

### Analysis on adjacent nucleotide dependence along ncRNAs

We conduct a statistical analysis to investigate if the dependence between the adjacent nucleotides in human ncRNA sequences can help to differentiate the false positives from the real ncRNAs. We consider two types of dependence: (1) the dependence of adjacent nucleotides inside the single-stranded region and (2) the dependence of adjacent base pairs inside the stacking pair region. For each family, we compare these two types of dependences in the known members with those in other candidates (most are suspected to be false positives) reported by Infernal to see if there is a significant difference which enables us to distinguish real ncRNAs from false positives.

After studying several possible methods to measure the dependence level between the adjacent nucleotide and the adjacent base pairs, we come up with an intuitive statistical measure for our study defined as follows. We obtain the multiple sequence alignment and the corresponding consensus secondary structure of all the members (i.e. full members) for each family from the Rfam database. For the candidates reported by Infernal, we deduce the multiple sequence alignment and the corresponding consensus secondary structure of them according to the alignment between each of these candidate sequences and the structure model reported by Infernal. Given a multiple sequence alignment 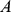 of a set of sequences, first considering the calculation of dependence between two adjacent columns 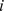 and 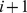 which are single-stranded in the consensus structure (single-stranded columns), let 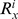 be the observed ratio of nucleotide 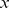 appearing in column 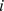 and 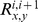 be the observed ratio of nucleotide 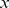 and 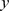 appearing in position 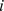 and 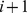. Based on probability theory, if there is no dependence between nucleotide 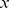 and 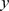 in column 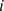 and 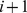, respectively, the value of 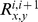 would be more or less the same as the product of 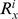 and 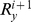. The value 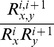 would capture the dependence of the nucleotides 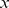 and 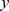 in column 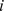 and 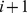. Since there are 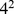 possible cases (i.e. 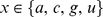 and 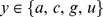), we compute an average of the values. So, we define the value of dependence level (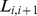) between the 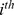 column and the 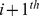 column as:
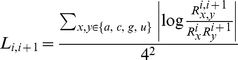
(1)


If there is no adjacent nucleotide dependence for columns 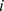 and 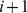, 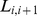 would tend to zero. A larger value of 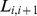 would imply the higher dependence level between the 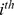 column and the 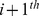 column. Let 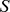 be the set of pairs of adjacent single-stranded columns (i.e. 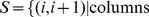 *i*, *i*+1 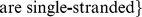). The level of dependence (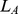) along all single-stranded regions in the consensus structure for the set of multiple sequence alignment 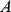 is defined as the average of the value of adjacent dependence level in all pairs of adjacent single-stranded columns along 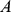:
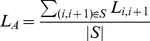
(2)


Similarly, to calculate the level of dependence between two adjacent base pairs, for two adjacent pairs of columns 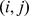 and 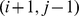 forming base pairs in consensus structure (paired column), let 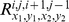 be the observed ratio of 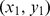 and 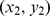 appearing in positions 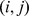 and 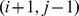. Then the value of dependence level (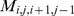) between the adjacent paired columns 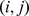 and 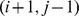 is defined as:
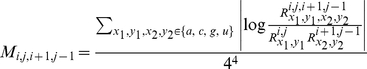
(3)


Let 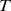 be the set of pairs of adjacent paired columns, the level of dependence (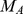) along all stacking pair regions in the consensus structure for the set of multiple sequence alignment 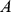 is defined as:
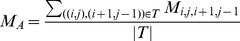
(4)


Larger value of 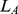 (or 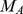) indicates the higher level of dependence between the adjacent single-stranded columns (or the adjacent paired columns) within the multiple sequence alignment 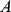. The comparison of the 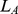 and 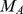 between all known ncRNA members (i.e. full members) in Rfam and the other candidates reported by Infernal of each family is shown in Table 1. The table lists the top 20 families with the highest number of candidates reported by Infernal. As we can see, in most of the cases, the level of adjacent dependence along both single-stranded regions and stacking pair regions in known ncRNAs is higher than that in other candidates reported by Infernal. The % of difference between the dependence levels with respect to the values of other candidates are 164% and 109% along single-stranded regions and stacking pair regions respectively. So, in the following, we try to make use of the adjacent nucleotide dependence in human ncRNA molecules to distinguish real ncRNAs from false positives.

### Filtering candidates from Infernal based on adjacent nucleotide dependence

Existing SCFG model does not consider adjacent nucleotide dependence. We propose a new order-1 SCFG model to incorporate this dependence (both the adjacent dependence of single bases and adjacent dependence of base pairs) in the model. The details of the model and how to construct the model (in particular, the production rules) from a family of ncRNA sequences will be given in “Materials and Methods” Section. In this section, we present the performance of using our new model to filter candidates from Infernal. For each candidate reported by Infernal, we go through our order-1 SCFG model which will give a score indicating the likelihood of the candidate being a member of the family. Since the number of parameters inside the order-1 SCFG model are much more than Infernal SCFG model and we used seed member (i.e. a small set of reliable known members) in Rfam 9.1 to build the model, in order not to overfit the model, we only consider the family with at least 30 seed members in the experiment.

In general, setting a threshold to distinguish real members from false positives is not a trivial task. Setting the threshold too high may decrease the sensitivity while a low threshold may include many false positives. However, using our order-1 SCFG model, from some empirical results (see below for details), the gap between the scores for real ncRNA members and those of false positives is large. This enables us to pick a threshold easily to ensure that all known members will not be considered as false positives. This also makes sure that we maintain the same sensitivity as Infernal, that is, all known members that can pass through SCFG model will also pass through our order-1 SCFG model. Moreover, the suggested thresholds listed in Rfam 9.1 was designed for the previous version of Infernal (v. 0.72) which is much slower compared with the new version of Infernal (v. 1.0) we used. Thus, using those thresholds for Infernal (v. 1.0) may not be appropriate. In order to have a fair comparison between Infernal and order-1 model, we pick three thresholds for Infernal and order-1 SCFG model respectively as follows. For each family, let the lowest score resulted by Infernal (or order-1 SCFG model) of all the known members (of all species) in the Rfam database be 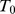 (or 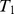). Setting the threshold to be 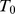 (or 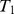) would give the full power of eliminating false positives, but without omitting any existing real members. We define 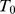 (or 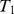) as an optimal threshold (opt-thres) for Infernal (or order-1 model). Yet to include possible novel members whose scores are lower than 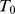 (or 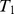), we also select two thresholds: 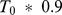 and 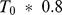 (or, 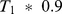 and 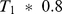) to compare the robustness of the filtering power of Infernal and our models. We select the families with at least 30 seed members. After using Infernal to identify the ncRNAs along all the human chromosome, we found that Infernal performs well and exhibits high sensitivity. From now on, we refer the candidates that reported by Infernal but not found in known databases as *false positives* for ease of discussion. Note that some of these candidates should be true positives, but the majority of them are probably false positives. As we can see in Table 2, the new order-1 SCFG model is able to filter more than 50% of those false positives reported by Infernal in all three thresholds. For the other families which are not shown in the list, the order-1 model can also filter over 50% of the false positives.

**Table 2:** Detailed filtering power of order-1 SCFG model.

		Opt		Opt*0.9		Opt*0.8	
Family	False positives	Infernal	Order-1	Infernal	Order-1	Infernal	Order-1
RF01382	8985	3633	1667	8985	2314	8985	3211
RF00017	4704	4704	1124	4704	1359	4704	1652
RF00037	1552	1435	559	1552	682	1552	788
RF00825	1415	0	0	0	0	3	0
RF00711	1019	68	12	139	23	308	35
RF00736	1012	696	288	1012	343	1012	412
RF00651	913	4	1	15	3	54	9
RF00647	906	23	0	64	0	170	0
RF00464	887	391	83	750	93	887	109
RF00031	687	687	256	687	302	687	357
RF00876	613	613	53	613	66	613	82
RF00693	548	22	27	99	42	417	62
RF00951	521	520	518	521	520	521	520
RF00131	485	0	0	0	0	0	0
RF00001	287	287	280	287	278	287	274
RF00646	270	2	0	7	0	38	0
RF00027	247	11	1	38	2	154	2
RF00685	243	3	0	4	0	10	0
RF00239	192	19	32	53	32	152	36
RF00140	190	0	4	0	6	14	6
**Total**	**25676**	**13118**	**4905**	**19530**	**6065**	**20568**	**7555**

For each family, let the lowest score resulted by Infernal (or order-1 SCFG model) of all the known members (of all species) in the Rfam database be 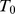 (or 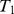). Setting the threshold to be 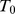 (or 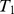) would give the full power of eliminating false positives, but without omitting any existing real members. We define 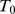 (or 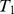) as an optimal threshold (Opt) for Infernal (or order-1 model). Yet to include possible novel members whose scores are lower than 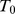 (or 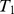), we also select two thresholds: 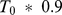 and 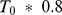 (or, 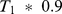 and 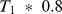) to compare the robustness of the filtering power of Infernal and our models. This table shows the detailed filtering power of order-1 SCFG model for the top 20 families in which Infernal reports the most number of false positives originally. As we can see in the table, the new order-1 SCFG model is able to filter more than 50% of those false positives reported by Infernal in all three thresholds. For the other families which are not shown in the list, the order-1 model can also filter over 50% of the false positives.

Since there is no other method to evaluate whether a candidate is indeed an ncRNA or a false positive unless laboratory approach is used, we use another popular software RNAz [8] to further evaluate the candidates filtered by our order-1 method. Only 3% are estimated to be ncRNAs by RNAz (see Table 3). We perform a similar test on the candidates that kept by our order-1 method. About 18–33% are estimated to be ncRNAs by RNAz. Although RNAz may not give an accurate result, it provides some evidence that most of the filtered candidates may be false positives. On the other hand, the candidates that kept by our order-1 method but cannot be confirmed by RNAz should be further evaluated.

**Table 3:** Use RNAz to further verify whether any of those false positives filtered by order-1 method and those not filtered by order-1 method is ncRNA.

		Filtered by order-1			Not filtered by order-1		
Threshold	False positives	Estimated as RNA	Total	%	Estimated as RNA	Total	%
Opt	13578	221	8201	2.7%	1795	5377	33.4%
Opt * 0.9	20661	383	13708	2.8%	1633	6953	23.5%
Opt * 0.8	22431	417	13902	3.0%	1586	8529	18.6%

We use another popular software RNAz [8] to further evaluate the candidates filtered by our order-1 method. Only 3% are estimated to be ncRNAs by RNAz. We perform a similar test on the candidates that kept by our order-1 method. About 18–33% are estimated to be ncRNAs by RNAz. Although RNAz may not give an accurate result, it provides some evidence that most of the filtered candidates may be false positives. On the other hand, the candidates that kept by our order-1 method but cannot be confirmed by RNAz should be further evaluated.

We remark that we have conducted another experiment to further justify that many of the candidates reported by Infernal are likely to be false positives. We generated a set of random sequences based on the human chromosomes as follows. We first checked all the existing short repeated sequences (with length less than 10) along the real chromosomes and then placed these set of repeated sequences in the same positions on the artificial sequence. Then for the rest of the positions, along every region of length 1000, we randomly generate the nucleotides with the same percentage of composition of different types of nucleotides (i.e. A, C, G, T) as the corresponding region in the real chromosome. We used Infernal scan through all the sequences and check how many candidates Infernal would report. Since it is unlikely to have a lot of real ncRNAs in these randomly generated sequences, so if Infernal still output a lot of candidates, many of them are likely to be false positives. Table 4 shows the result of Infernal and the proposed order-1 SCFG model on the simulated artificial sequence. As one can see, Infernal still reports quite an amount of regions as potential RNAs. The order-1 model also can filter out around 50% of them.

**Table 4:** Result of Infernal and our order-1 SCFG model on simulated data.

		Opt		Opt*0.9		Opt*0.8	
Family	False positives	Infernal	Order-1	Infernal	Order-1	Infernal	Order-1
RF01382	14174	7115	2915	14174	4022	14174	5262
RF00037	797	734	315	797	371	797	430
RF00031	435	435	114	435	136	435	168
RF00559	316	25	0	112	0	316	0
RF00736	243	169	97	243	116	243	134
RF00693	179	8	9	26	18	139	24
RF00876	150	150	2	150	4	150	7
RF00825	86	0	0	0	0	0	0
RF00711	82	3	6	6	7	12	11
RF00239	73	4	15	10	16	55	16
RF00661	72	60	54	72	54	72	55
RF00379	59	0	0	2	0	16	0
RF00464	52	4	4	34	5	52	6
RF00001	44	43	37	44	34	44	30
RF00651	42	0	0	0	0	2	0
RF00188	39	1	0	4	1	24	2
RF00614	37	5	0	16	0	37	0
RF00131	36	0	0	0	0	0	0
RF00519	35	14	0	35	0	35	0
RF00468	30	0	0	0	0	4	0
**Total**	**16981**	**8770**	**3568**	**16160**	**4784**	**16607**	**6145**

### Gap between scores of known members and false positives

**Figure 1:**
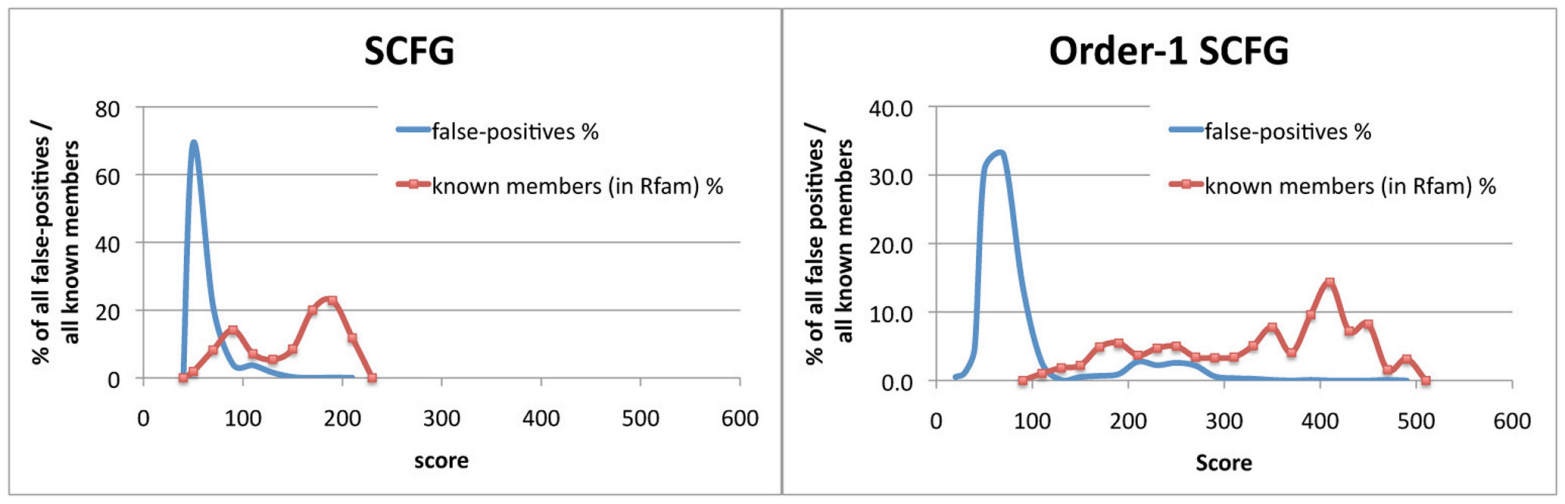
Comparison of the score distribution between false positives and all the known members (in Rfam) of the family RF00017 based on SCFG and order-1 SCFG model.

The improved filtering power is due to the fact that our order-1 SCFG model can enlarge the difference between the score of the false positives and that of the true positives. As shown in Figure 1, we select family RF00017 and plot the distribution of the scores of all the known ncRNA members in Rfam and the false positives based on the original SCFG model and our order-1 SCFG model. From the figure, we see that the average scores of the false positives and the true positives are 69.7 and 157.7 respectively for the original SCFG model while the corresponding scores are 104.6 and 344.6 respectively for the order-1 SCFG model. The difference between the scores of the false positives and the true positives is significantly larger in our order-1 SCFG model than that of the original SCFG model. The order-1 SCFG model seems to characterize real ncRNAs in a better way than the original SCFG model, thus have a better filtering power to remove the false positives.

## Materials and Methods

The following subsection will define the order-1 stochastic context free grammar and the next subsection will describe how to build the order-1 SCFG model to represent a set of ncRNA sequences.

### How to incorporate adjacent nucleotide dependence

In the SCFG model used in Infernal, the transition from a state to another state only depends on the current state without considering the current nucleotide. To capture the dependence of adjacent nucleotides, we introduce a new type of states labeled 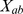 or 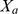. The state 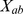 emits the pair of symbols 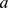 and 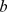 while 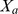 emits only symbol 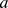. Therefore, the probability of which state to go depends on the current state, the current nucleotide(s), the state to go, and the nucleotide(s) it emits. The adjacent dependence thus has been captured. As the transition probability depends on the current and the next state as well as the nucleotides they emit, we refer it as an order-1 SCFG.

#### Formal definition

We define the order-1 SCFG as follows: Order-1 SCFG = 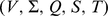 where

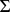
 is a finite set of terminal symbols. In RNA, there are four types of terminal symbols (i.e. A, C, G, U).

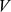
 is a finite set of non-terminals (which are called states here). There are seven types of states, which are 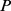, 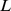, 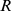, 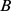, 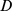, 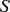 and 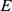. The description of each type is listed in Table 5. Let 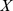 be any one of the types. Define 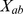 to be a type-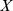 state emitting two terminal symbols 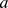 and 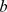, 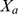 for emitting one symbol 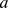, and 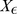 for emitting no symbol.

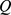
 is a set of production rules of the form: 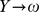 where 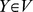 and 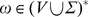

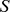
 is the start variable, where 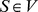

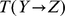
 is transition score from state 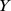 to state 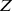 where 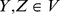. The value of transition score is defined based on the transition probability from state 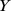 to state 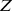.

**Table 5:** The set of production rules of Order-1 SCFG.

State type	Description	Production rule	Score
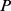	pair emitting	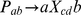	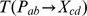
		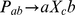	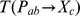
		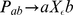	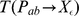
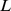	left emitting	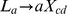	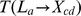
		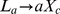	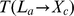
		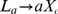	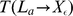
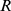	right emitting	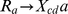	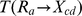
		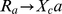	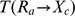
		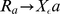	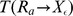
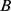	bifurcation	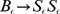	0
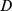	delete	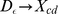	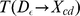
		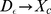	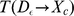
		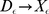	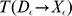
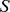	start	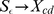	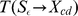
		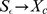	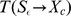
		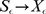	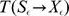
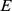	end	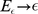	0

Note that P-type state indicates the pair-wise relationship between two symbols, so the grammar can produce not only a sequence of symbol, but also the corresponding structure of the sequence.

The production rules are listed in Table 5 and Table 6 shows an example of applying the production rules to generate a sequence with corresponding structure. Because P-type state indicates the pair-wise relationship between two symbols, the grammar can produce not only a sequence of symbol, but also the corresponding structure of the sequence.

**Table 6:** Example of applying production rules to generate a sequence with corresponding structure.

Position	1	2	3	4	5	6	7	8
Sequence	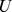	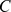	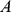	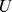	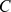	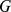	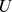	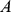
Structure		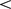	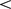	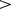		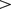	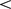	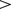

To generate the above sequence with corresponding structure, steps are:
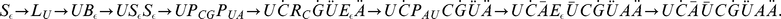

Note that the single dots, the bars and the double dots at the top of the characters indicate the corresponding base-pairs emitted by state 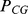, 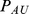 and 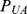.

Each production rule has its own transition score and the choice of production rule depends on both the current and the next emitted symbol(s). That means the transition score would be depends on the adjacent emitted symbol(s). Therefore this order-1 stochastic context free grammar can capture the dependence between the adjacent symbols (or called nucleotides for RNA sequence).

### Building an order-1 SCFG for an ncRNA family

In this subsection, we focus on building an order-1 SCFG model to represent a given ncRNA family. The model includes a set of states and edges, and each edge consists of a score. The set of states is the set of non-terminals 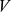 that appear in the production rules in the previous section and an edge connecting state 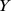 and state 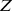 if there exists a production rule in which state 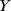 can be transited to state 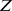. The score of the edge equals to the corresponding transition score between the two states.

By given a multiple sequence alignment of a set of member sequences of a ncRNA family and the corresponding consensus secondary structure, our aim is to build an order-1 SCFG model to represent the family. The consensus secondary structure is assumed to be regular and have no pseudoknot. It is suggested to use the multiple sequence alignment of the seed members and the consensus secondary structure provided by Rfam 9.1 as an input to build the order-1 SCFG model for the corresponding ncRNA family.

**Figure 2:**
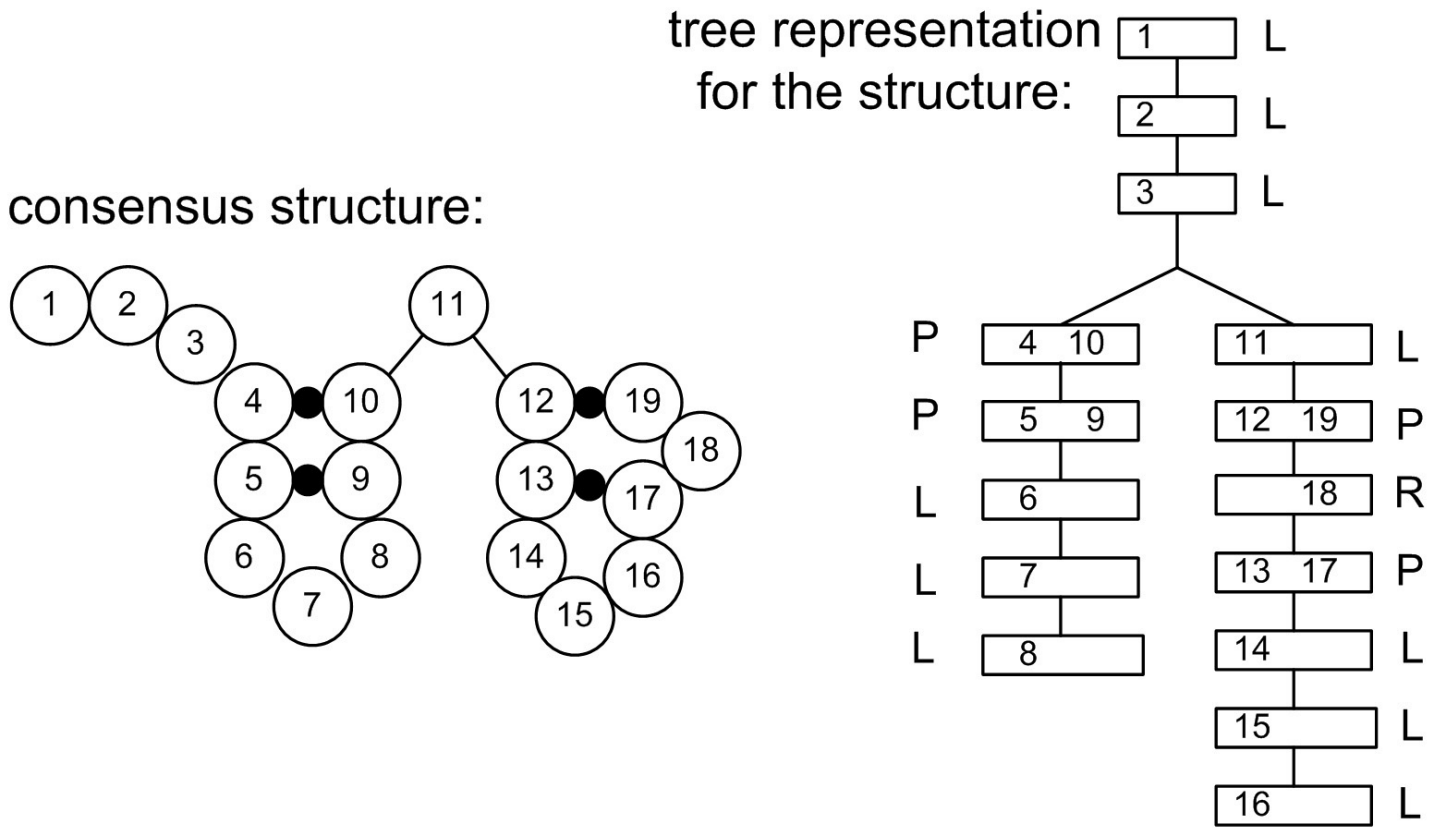
Left: Consensus structure. Right: Tree representation of the consensus structure.

Before building the order-1 SCFG model, we use a tree structure to represent the consensus structure according to the method stated in [9]. Figure 2 shows an example about the tree representation for a ncRNA structure. In the consensus structure, the black dots between the positions indicates the pairwise relationship between them. In the resulting tree representation, there are three kinds of nodes: L, R and P. The L-type and the R-type node represent a single base in the ncRNA structure while the P-type node represents a base-pair. The sequence of positions can be regenerated by a traversal of the tree from root to leaves and from left to right.

**Figure 3:**
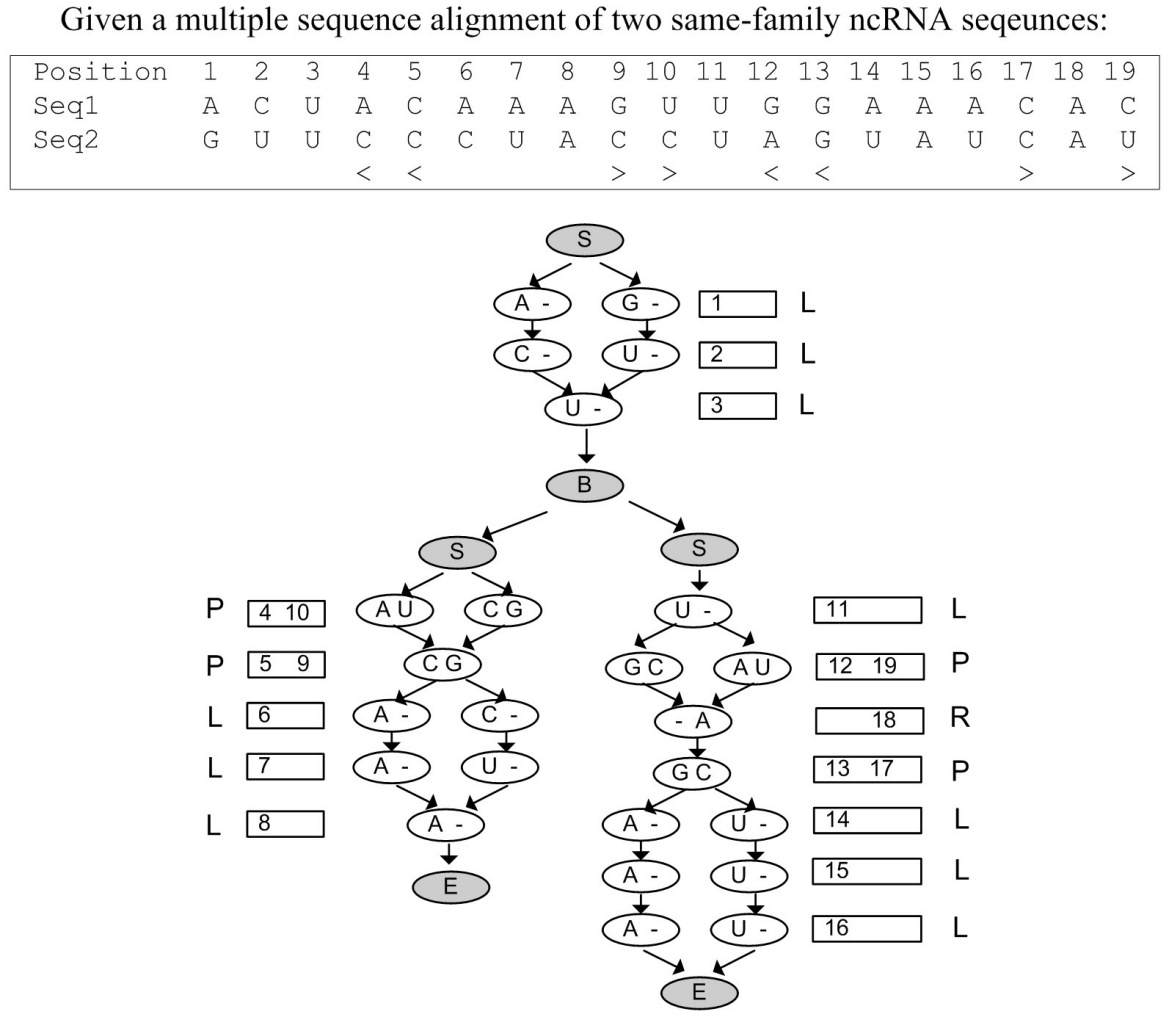
A high-level picture of an order-1 SCFG model for the multiple sequence alignment of two sequences.

In Figure 3, we use a multiple sequence alignment of two sequences and the consensus structure as an example to illustrate the high-level idea of order-1 SCFG model. The model is represented as a non-cyclic graph and the structure of the graph is according to the tree representation of the consensus structure of the input multiple sequence alignment. The circles indicate the states and the arrows represent the edges connecting from one state to another. The gray states do not represent any position in the structure and emit no symbol, while the white states either represent a single-base position in the structure and emit one symbol for that position, or represent base-pair positions and emit two symbols for the base-pair.

Consistent with the production rules, there are seven types of states: 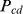 (pair emitting symbol 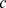 and 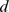), 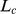 (left emitting 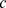), 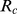 (right emitting 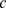), 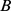 (bifurcation), 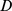 (delete), 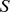 (start) and 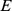 (end), where 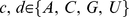. 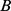-type state indicates a branch in the graph, representing also a branch in the structure. 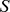 is the first state of the graph and of each branch, and 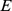 is the end state of each branch. 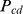 represents a base-pair and 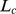, 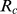 represents a single base in the corresponding positions. The type of white state is indicated beside the corresponding rectangular box. There may be more than one state in the same single-base position or base-pair positions, like 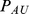 and 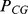 in (4,10), indicating more than one possible sets of symbols appearing in the base-pair positions. One may find that there exists a path (with branch) in the graph such that each of the two given sequence with the corresponding structure can be produced. As illustrated in the figure, the adjacent dependence can be captured in the graph. For example, if one choose to emit symbol 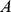 in the position 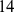, then symbol 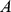 would also be the only choice for position 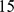 and 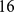.

**Figure 4:**
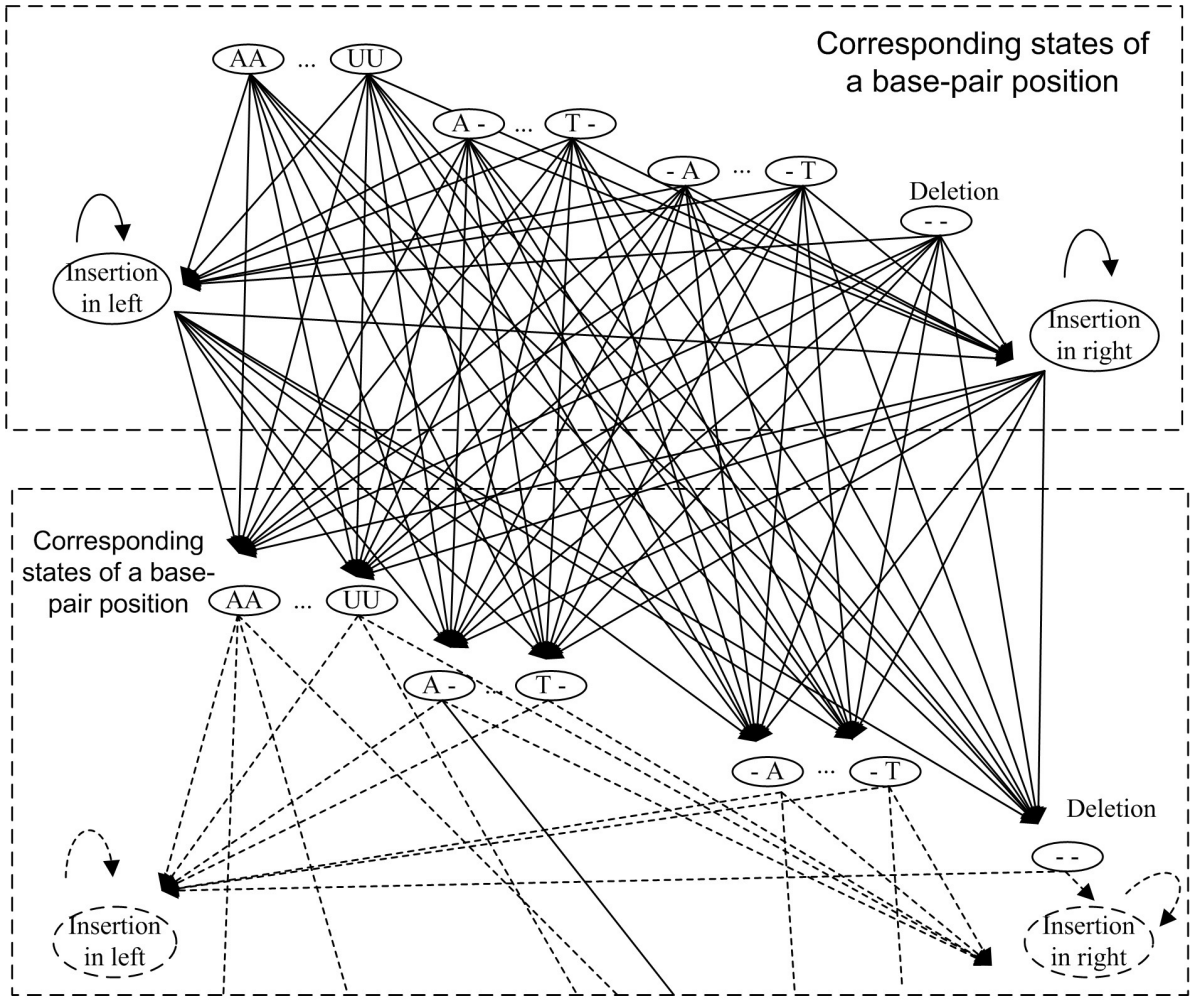
The details of order-1 SCFG model in two base-pair positions.

In order to check whether an input sequence is a member of a ncRNA family, the model should be able to identify any type of sequence. Therefore, the model should consider mutation, insertion and deletion in all the positions. The insertion state, deletion state and states of all possibilities should be included in each position. For example, in a single-base position, there should be four states representing four possibilities (i.e. A, C, G, U); in base-pair positions, the possibilities include (i.e. AA, AC, … , UG and UU). Figure 4 shows the details of order-1 SCFG model in two consecutive base-pair positions. The edges between the states indicate the possible choice of the states along the path and different sequence will be produced for each path. The resulting score of the path is the sum of the scores of all the edges passed through along the path. In the next subsection, we will mention about how to set up the score for each edge.

### Parameters setting

The purpose of the experiment is to evaluate whether after considering the adjacent dependence in the SCFG model, there would be any improvement in identifying the ncRNA family members. In order to make the result sensible and meaningful, we do not tune the parameters from scratch because it may change the condition of the entire model completely. We have to keep the weighting of the scores the same as that of the SCFG model in Infernal and we hope to make sure there is no other factor except the incorporating of the adjacent dependence which will affect the performance. In the following, we use *SCFG* to refer to the model in Infernal, and *order-1 SCFG* for our new model.

Therefore, we derived the set of parameters for order-1 SCFG model directly from the model built by Infernal mathematically. Before we describe how to derive the parameters from SCFG model, we first look into the method Infernal used behind to derive the parameters.

In the SCFG model, for each edge from the state 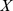 to the state 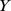, it has a transition score 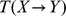. we can directly obtained the value of 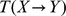 from the model built by Infernal. The equation of 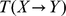 is as follows:
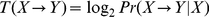
(5)where 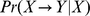 is the transition probability from state 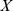 to state 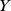 given 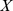 in SCFG.

For each state 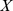 in SCFG, we can find a set of states 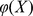 in order-1 SCFG such that each state 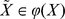 is a particular case of 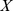. For example, if 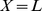, then 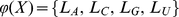, where 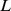, 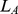, …, 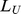 are defined in the previous section. For each transition from state 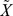 to state 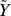 in order-1 SCFG model where 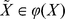 and 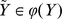,
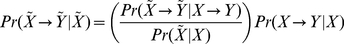
(6)


Equation 6 establishes the relationship between the transition probability from state 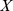 to state 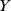 in SCFG, and the transition probability from state 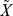 to state 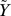 in order-1 SCFG where 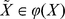 and 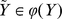. We can approximate the values of 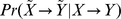 and 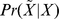 by the following equations:
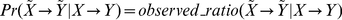
(7)

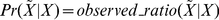
(8)where 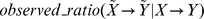 is the ratio of the traversal of 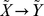 among the seed members (in Rfam 9.1, a small set of known reliable members are referred as seed members) under the condition of the traversal of 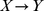. As mentioned in the previous section, the choice of the traversal of 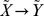 depends on the current and the next emitted character(s). Therefore, for example if 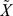 is 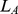 and 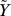 is 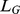, then the traversal of 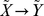 emits 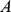 in column 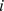 and emits 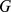 in the column 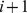, the 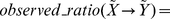
observed frequency of 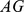 in column 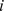 and 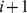 among the seed members divided by the total number of instances where the characters in both column 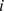 and column 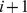 are not space (i.e. 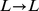). Precisely, we add 0.1 more for each possible case so that for those cases there is no instance, their probability would not be zero. Computation of 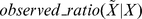 is similar.

Consider a production rule (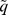) which transverse from state 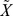 to state 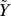 in order-1 SCFG and will emit another set of symbol(s) 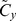 at state 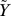, where 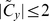. According to the formula used inside the Infernal, the score of the production rule score(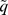) should be defined as follows:
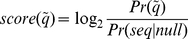
(9)

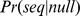
 is the corresponding probability for a random-sequence model. Infernal does not consider any bias among different type of nucleotides, and so consider the probability for each type of nucleotide is 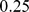. Therefore, to calculate 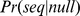 for the production rule 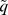 for the traversal of 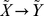, since when the production rule is applied it is assumed to be in the state 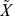, we should consider the emitted symbol(s) in the next state, 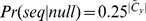. Thus, by Equations 5,6,7,8 and 9, we set the transition score of each production rule 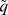 in order-1 SCFG as:
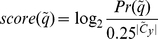
(10)

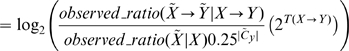
(11)


### Other details

To check whether there exists any region inside the sequence such that the region is probably the member of a specific ncRNA family, we can perform alignment between the order-1 SCFG model of the family and the sequence. If there is a region such that the resulting alignment score is high, then we can regard the region as a potential member of the family.

A dynamic programming algorithm was introduced to align a SCFG model with a sequence [9]. [11] also suggested another memory-efficient algorithm which can further decrease the memory usage. All of these methods can be applied to the order-1 SCFG model directly. The time complexity of the alignment algorithm is 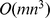, where 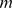 is the length of the input sequence and 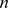 is the number of states inside the model. Although the number of states in order-1 SCFG model is more than the number of states in SCFG model, many of states in fact can be skipped when calculating the score during the dynamic programming. For example, if we are mapping 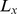 where 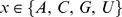 states to a position of the input sequence and the nucleotide in that position is 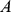, then we do not need to consider the states like 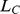, 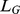 or 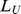, but only consider 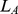. The total number of states required to scan through when calculating the scores for each position of the sequence is the same. Therefore the time required for aligning an order-1 SCFG model with a sequence is also the same.

Regarding the details of the experiment, the version of the human chromosomes downloaded from NCBI website is 36.3. And the version of the Infernal software is 1.0. We downloaded all the multiple sequence alignments of the seed members and full members of all the ncRNA families from the Rfam 9.1 database. For each family we used the corresponding multiple sequence alignment of the seed members to build the models by using Infernal with default parameters. When we performed scanning on the human chromosomes, we executed the search program of Infernal with an option ‘–ga’, which would perform filtering according to the family-specific thresholds suggested in Rfam 9.1. Regarding the small computer cluster, we use four nodes and each node has 2×core 2 quad CPU 2.4GHz with 32G memory. By using the cluster, it takes around 1.5–2 weeks to finish scanning one human chromosome for all around 1300 families. Regarding the verification step using RNAz, we divided all the false positives into two clusters - those filtered by order-1 method and those not filtered by order-1 method. For each cluster, we computed the corresponding multiple sequence alignment of the false positives according to the resulting alignments outputted by Infernal and divided them into groups of six as inputs to RNAz (since at most 6 sequences can be inputed to RNAz each time) for ncRNA determination.

### Concluding remarks

In this work, we showed that using dependency of adjacent nucleotides in ncRNAs can improve the accuracy of identifying ncRNAs and we developed a new order-1 SCFG model to capture this dependency for identifying ncRNAs. The results are promising. There are a few issues to be further investigated. It is possible that the adjacent nucleotides in ncRNAs may show a more significant dependence in regions (such as stems which are more related to the functionalities of a ncRNA) than other regions in the same ncRNA. A more detailed analysis can be carried out to investigate along this direction and further improve the model to capture this characteristics. Also, the current order-1 SCFG model may not perform very well for families with only a few seed members which may overfit the model. To make it work well for these families may be desirable. To conclude, we believe that with more in-depth investigation on nucleotide dependency on ncRNAs, we may be able to come up with better computational approach for identifying ncRNAs.
